# Brazilian National Policy of Comprehensive Women’s Health Care and mortality during climacteric period: has anything changed?

**DOI:** 10.1186/s12889-021-10556-8

**Published:** 2021-03-16

**Authors:** Isabel Cristina Esposito Sorpreso, Francisco Winter dos Santos Figueiredo, José Lucas Souza Ramos, Lea Tami Suzuki Zuchelo, Fernando Adami, Edmund Chada Baracat, José Maria Soares Júnior

**Affiliations:** 1 Disciplina de Ginecologia, Faculdade de Medicina (FMUSP), Universidade de São Paulo, Dr. Enéas de Carvalho Aguiar Avenue, 255, 10° andar, sala 10.166 – CEP: 05403-000, São Paulo, SP Brazil; 2 Laboratório de Epidemiologia e Analise de Dados, Centro Universitário Saúde ABC (CUSABC), Santo André, São Paulo Brazil; 3 Escola Superior de Ciencias da Santa Casa de Misericordia de Vitoria (EMESCAM), Vitoria, Espírito Santo Brazil

**Keywords:** Menopause, PNAISM, Brazil, Women’s health, Climacteric

## Abstract

**Background:**

The National Policy for Integral Attention to Women’s Health Care (PNAISM) was implemented in 2004, with monitoring of potential benefits. One of the life cycles of women contemplated in this health policy was the importance of health care during the climacteric. Prevention and health promotion are actions carried out by the Brazil National Health System and enshrined in health Brazilian policies for women. Thus, our purpose was to identify climacteric women’s main causes of death as well as the mortality trends of such causes, especially after implementation of PNAISM.

**Methods:**

An ecological study was conducted from 2018 to 2020. Data were retrieved from the Brazilian Health Department by accessing the mortality information system of the National Health Information, divided into periods 1996–2004 and 2005–2016 the latter to correspond with the implementation of the National Policy. The death records of Brazilian women aged 40 to 64 years who had a designated cause of death were retrieved. Trends and differences between periods were evaluated using linear regression. The significance level was set at 5%.

**Results:**

The main causes of death in women from 1996 to 2016 were circulatory system diseases (22.47%, 697,636 deaths), neoplasms (19.69%, 611,495 deaths), respiratory system diseases (5.5%, 170,716 deaths), endocrine, nutritional, and metabolic disorders (5.27%, 163,602 deaths), and digestive system diseases (3.74%, 116.280 deaths). Analyzing the changes in the major causes of death of climacteric women after implementation of the PNAISM we observed that mortality from circulatory system diseases and endocrine and nutritional diseases were significantly declined in post-PNAISM period: (β = − 3.63; 95% CI – 4.54 to − 2.73 *r*^2^ = 0.87; *p* < 0.001; β = − 0.51; 95% CI, − 0.71 to − 0.31; *R*^2^ = 0.73; *p* < 0.001, respectively). No changes were observed in mortality from neoplasms and respiratory system diseases in post-PNAISM period (*p* = 0,765; *p* = 0,233, respectively).

**Conclusions:**

After implementation of the PNAISM, we observed a downward trend in rates of mortality from diseases of the circulatory and digestive systems and from endocrine, nutritional, and metabolic diseases but stability in the rates of death from neoplasm and respiratory system diseases.

## Background

The National Policy of Comprehensive Women’s Health Care – “Programa Nacional de Atenção Integral à Saúde da Mulher” (P NA I S M), launched by the Brazilian Health Department in 2004 and presently operating in conjunction with the Unify Health System (UHS) - Sistema Único de Saúde (SUS), aims to reduce women’s morbidity and mortality, especially deaths resulting from preventable causes in all life cycles [1–3].

Historically, the assistance and care provided to women by the Brazilian health system was restricted to pregnancy and the puerperium, and health actions were specific (vertical) and oriented to maternal and child health [1–3]. In 1984, with the creation of the “Programa de Atenção Integral à Saúde da Mulher” - PAISM (Program for Integral Assistance to Women’s Health) and the implementation of SUS, differentiated attention to women’s health was initiated, related to longitudinal assistance, in addition to other aspects relevant to the health of the female population, such as prevalent gynecological diseases; prevention, detection, and treatment of neoplasms; menopause; domestic and sexual violence; and the incorporation of sexual and reproductive rights at different levels of complexity [1–4].

In 2004, PAISM was restructured with new guidelines and goals for assisting women’s health, known as the National Policy for Comprehensive Care for Women’s Health (PNAISM), and priority was given to guarantee health care for women in Brazil during the climacteric period [1–3]. The climacteric is the transition period in a woman’s life from the potential for reproducing to a nonreproductive phase [5]. At such a time, clinical changes occur in association with prolonged, permanent, and physiological hypoestrogenism [5–7], and they may be related to the onset or aggravation of chronic noncommunicable diseases [8, 9]. In this period, ranging from age 40 to 65 years, a woman can benefit from health prevention and promotion actions taken by the SUS and PNAISM.

The epidemiological mortality profile in Brazil has a marked prevalence of chronic noncommunicable diseases and shows a tendency over recent years toward reduction in the deaths, specifically of climacteric women, of the circulatory system [8] and ill-defined causes as well as a trending increase in neoplasms [8–10].

Studies conducted in recent years on the impact of PNAISM on women’s health and on the trends in the causes of disease and death among menopausal women have been scarce [8–10]. Studies on the mortality of Brazilian women may contribute to changes in health promotion strategies and to the monitoring of the organization and implementation of public health care policies [11, 12]. Thus, our purpose was to identify the main causes of death in climacteric women as well as the time trends in such cause-specific mortality, especially after implementing PNAISM.

## Methods

### Study design

Secondary data analyses were performed using the Setor de Atenção Primária, Disciplina de Ginecologia, Departamento de Obstetrícia e Ginecologia, Faculdade de Medicina, Universidade de São Paulo from 2018 to 2020. Health information is centralized in the official database of the Brazilian Health Department with data managed and supplied by the IT Department of SUS (DATASUS) and by the Brazilian Institute of Geography and Statistics (Instituto Brasileiro de Geografia e Estatística [IBGE]), which is the main office responsible for sociodemographic data in Brazil. In this study, data were obtained from DATASUS through access to the Systems of Information on Mortality (Sistemas de Informação sobre Mortalidade) (SIM) for retrieval of vital statistics indicators and through access to the System of Health Information (Sistema de Informações de Saúde [TABNET]) for the extraction of demographic and socioeconomic indicators.

### Data source

The DATASUS database covers approximately 96% [13] of the Brazilian population and provides socioeconomic data and information related to health and health care. The data made available by DATASUS are computerized records of procedures, actions, and services performed by SUS. The data go through an internal validation process before being made available for free public access. However, the individuals treated at SUS are not identified by name, rendering more recent data on the general health of the population vulnerable to subsequent alterations. Hence, they were not utilized for analysis.

### Participants

Participants were selected from the databases. Only the records of women who died between 40 and 64 years of age—but not during pregnancy or the postpartum period—who were residents of Brazil and who had a designated cause of death were retrieved. This age range was established based on the definition of climacteric by the World Health Organization [14]. All deaths occurring in this population between 1996 and 2016 that were reported to the SIM were included in this study.

### Variables

#### Age patterns of mortality

Data on causes of death were retrieved according to the tenth edition of the International Classification of Diseases (ICD-10) [15]. Women who died between 40 and 64 years of age had their records retrieved and were stratified into 4-year age brackets (40–44, 45–49, 50–54, 55–59, and 60–64 years) and year of death.

The estimates of the resident population were obtained from two projections made by IBGE [10]. The population estimates for the years between 1996 and 2000 was based on the intercensal projections covering the years between 1981 and 2012 and was organized by age bracket, sex, and housing status, and between 2001 and 2015 from projections for the 2000 to 2006 period, the population was classified by sex and age. The resident population was arranged into 4-year age brackets.

Mortality rates were calculated as the number of deaths among women per 100,000 women resident in the population. The resulting rate was age-standardized using the direct method, based on the population in the world as a reference [16].

#### Periods related to PNAISM

Rate behavior was analyzed according to ranges related to the year of PNAISM implementation. The pre-implementation period corresponds to the 1996 to 2004 range and the post-implementation period to the subsequent 2005 to 2016 range.

### Ethical considerations

This study analyzed secondary data freely accessible to the public and thus respects work ethics and upholds good research practices. As a result, the work cannot present any approval number given by the research ethics committee. According to resolution number 510 of the National Health Council published on April 5, 2016, studies conducted with secondary data or with public data that render identification of the individual impossible are not required to be submitted for approval or evaluated by the research ethics regulatory system in Brazil, be it the Research Ethics Committee or the National Council on Research Ethics [17].

### Data analysis

Frequency distributions of causes of death, using the ICD-10 categories, was performed with absolute numbers and frequency relative to the number of deaths documented during the period. Trends were evaluated by linear regression, and the slope (B) and the respective 95% confidence interval, the explained variance (*R*^2^), and the probability value (p) for each cause of death were calculated. All mortality rates were unadjusted for any potentially confounding variables. The significance level was set at 5%. Stata 11® (StataCorp, LCC) was used.

## Results

Deaths from non-obstetric causes of women aged 40 to 64 years totaled 2,107,634 between 1996 and 2016. The five major causes of death were classified as circulatory system diseases, neoplasms, respiratory system diseases, endocrine, nutritional, and metabolic disorders, and digestive system diseases totaling more than half (56.67%) of the deaths in that period (Table 1).

**Table 1:** Distribution of deaths of climacteric women residents of Brazil, between 1996 and 2016 according to the tenth revision of the International Classification of Diseases (ICD-10)

Classification	Section (ICD)	Deaths	%
Diseases of the circulatory system	IX	697,636	22.47%
Neoplasms (tumors)	II	611,495	19.69%
Diseases of the respiratory system	X	170,716	5.50%
Endocrine, nutritional, and metabolic diseases	IV	163,602	5.27%
Diseases of the digestive system	XI	116,280	3.74%
Some infectious and parasitic diseases	I	112,459	3.62%
External causes of morbidity and mortality	XX	108,039	3.48%
Diseases of the genitourinary system	XIV	45,897	1.48%
Diseases of the nervous system	VI	28,079	0.90%
Mental and behavioral disorders	V	13,728	0.44%
Diseases of the musculoskeletal system and connective tissue	XIII	13,290	0.43%
Blood and hematopoietic organ diseases and some immune disorders	III	13,231	0.43%
Diseases of the skin and subcutaneous tissue	XII	5469	0.18%
Congenital malformations, deformations, and chromosomal abnormalities	XVII	4253	0.14%
Diseases of the ear and mastoid process	VIII	310	0.01%
Diseases of the eye and adnexa	VII	55	0.002%

*ICD-10* International Classification of Diseases-10

**Fig. 1:**
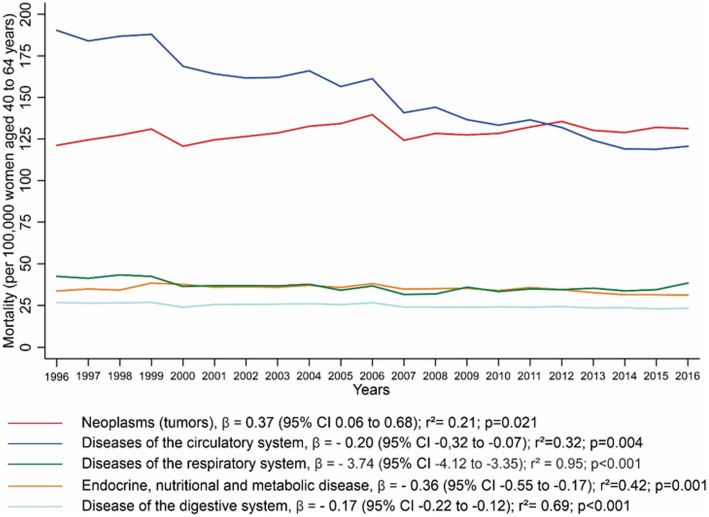
Mortality trend of the main causes of death in climacteric Brazilian women between 1996 and 2016

Figure 1 shows the trend analysis of the five major causes of death from 1996 to 2016, and it showed a significant reduction in mortality rates from diseases of the circulatory system (β = − 0.2; 95% CI, − 0.32 to − 0.07; *R*^2^ = 0.32; *p* = 0.004), respiratory system (β = − 3.74; 95% CI, − 4.12 to − 3.35; *R*^2^ = 0.95; *p* < 0.001), endocrine, nutritional, and metabolic diseases (β = − 0.36; 95% CI, − 0.55 to − 0.17; *R*^2^ = 0.42; *p* = 0.001) and digestive system (β = − 0.17; 95% CI, − 0.22 to − 0.12; *R*^2^ = 0.69; *p* < 0.001) as well as an increase in mortality from neoplasms (β = 0.37; 95% CI, 0.06 to 0.68; *R*^2^ = 0.21; *p* < 0.021) (Fig. 1). From these results we observed a relatively low R-squared values for mortality from neoplasm and circulatory system diseases (*R*^2^ = 0.21 and *R*^2^ = 0.32, respectively).

Mortality from circulatory system diseases significantly declined in both periods (pre-PNAISM: β = − 3.94; 95% CI − 5.83 to − 2.06 *r*^2^ = 0.77; *p* = 0.002) and Post-PNAISM: β = − 3.63; 95% CI – 4.54 to − 2.73 *r*^2^ = 0.87; *p* < 0.001). Mortality from endocrine and nutritional diseases did not change in the pre-PNAISM period (*p* = 0.138); however, it began to decline in the post-PNAISM period (β = − 0.51; 95% CI, − 0.71 to − 0.31; *R*^2^ = 0.73; *p* < 0.001). No changes were observed in mortality from neoplasms in both periods (pré-PNAISM: *p* = 0,114 and post-PNAISM: *p* = 0,765) (Table 2).

**Table 2:** Changes in the major causes of death of climacteric women during pre-implementation and post-implementation of the National Policy of Comprehensive Women’s Health Care

Sections	Pre-PNAISM 1996 to 2004			Post-PNAISM 2005 to 2016		
	β (95% CI)	*R*^2^	p*	β (95% CI)	*R*^2^	p*
Diseases of the circulatory system	- 3.94 (− 5.83 to − 2.06)	0.77	0.002	−3.63 (−4.54 to − 2.73)	0.87	< 0.001
Neoplasms (tumors)	0.84 (− 0.25 to 1.94)	0.22	0.114	−0.11 (− 0.90 to 0.69)	0.01	0.765
Diseases of the respiratory system	−0.85 (−1.44 to − 0.26)	0.57	0.011	0.20 (− 0.15 to 0.54)	0.05	0.233
Endocrine, nutritional, and metabolic diseases	0.30 (−0.12 to 0.74)	0.18	0.138	−0.51 (− 0.71 to − 0.31)	0.73	< 0.001
Diseases of the digestive system	− 0.13 (− 0.39 to 0.14)	0.04	0.287	− 0.22 (− 0.33 to − 0.10)	0.59	0.002

*95% CI* 95% confidence interval, *R*^*2*^ explained variance, *β* Slope, *PNAISM* Política Nacional de Atenção Integral à Saúde da Mulher (National Policy of Comprehensive Women’s Health Care)

*Linear regression

Additionally, mortality rates for diseases of the respiratory system declined over the pre-PNAISM period (β = − 0.85; 95% CI, − 1.44 to − 0.26; *R*^2^ = 0.57; *p* = 0.011) but did not change significantly in the post-PNAISM period (β = 0.20; 95% CI, − 0.15 to 0.54; *R*^2^ = 0.05; *p* = 0.233), and the non-significant decrease of the mortality of diseases of the digestive system in pré-PNAISM, changes to the significant decrease on the period post-PNAISM (β = − 0.22; 95% CI, − 0.33 to − 0.10; *R*^2^ = 0.59; *p* = 0.002) (Table 2).

## Discussion

In this study, the diseases of the circulatory and respiratory systems as well as endocrine, nutritional, and metabolic diseases and digestive system diseases accounted for approximately 37% of the deaths of women in the 40 to 64 year age bracket, while neoplasms alone were responsible for almost 20% of women’s deaths in that same population.

The convergence of proposals originating from the Brazilian National Health System over these years as well as the development of public health policies for women, such as the Comprehensive Assistance Program for Women’s Health (PAISM) in 1984, and its new guidelines in 2004 represent a milestone in the history of public policies aimed at women, breaking the traditional perspective of maternal and child life with the advent of the importance of prevention and health promotion for non-transmissible chronic diseases [1, 2, 4, 18].

The discussion about public policies and climacteric women emphasizes the importance of preventing damage and promoting health, considering that the life cycle is mainly associated with hormonal instability and the reduction of estrogen levels that may affect mortality due to cardiovascular diseases and neoplasms. However, the unavailability of health services interferes with the demand of women in the climacteric period to receive guidance and resolution on issues that affect that life stage [19–23].

In addition, the authors recommended the need to put into practice the constitutional right to integrality in health and to contemplate health promotion, and prevention in Brazilian health policies. This achievement must preserve women’s rights and expand assistance services, so that they can correspond, quantitatively and qualitatively, to the demands of female users of the Brazilian health system [24, 25].

The epidemiological profile of mortality in Brazil currently shows a marked predominance of circulatory system diseases and neoplasms as agents of death since 1985. In that year, such causes overrode infectious and parasitic diseases and became the chief agents of death in the country [26, 27]. This tendency, specifically in women in the climacteric, was demonstrated by Schmitt et al. [8], who found, in decreasing order of magnitude of mortality rates, circulatory system diseases, neoplasms, symptoms, signs, and ill-defined disorders, respiratory system diseases, external causes (external causes of morbidity and mortality among women aged 40–65 years), diseases of the digestive system, infectious and parasitic diseases, endocrine, nutritional, and metabolic diseases, genitourinary system diseases, and nervous system diseases.

In our study, 56,7% of the deaths among women aged 40–65 years that have occurred in the last 20 years were caused by circulatory system diseases, neoplasms, respiratory system diseases, endocrine, nutritional, and metabolic diseases, and digestive system diseases.. The death distribution shown by the categorizations of the tenth International Classification of Diseases is similar to the distribution of deaths among women found in the results of national studies [8–11].

The higher percentages of deaths due to cardiovascular diseases, neoplasms, and metabolic diseases constitute a familiar scenario to women in the age bracket corresponding to post menopause [27, 28], and they are attributed to factors related to lifestyle, such as smoking, alcoholism, excess weight, and high blood pressure [28]. These are frequent habits and concomitant diseases reported during postmenopause and may occur or aggravate as a result of physiological and progressive hypoestrogenism [27, 28].

The downward slope of the mortality rates of cardiovascular and metabolic diseases and an increase in mortality due to neoplasms were observed in a study by Mondul et al. [6], who found that mortality from circulatory and ill-defined diseases was declining and that for neoplasms was moving upward.

One of the guidelines of PNAISM is related to health care in all of women’s life phases and mainly to the circumstances that aggravate health, but can be avoided, prevented, or detected early on, such as breast and cervical cancers [1–4].

Implementation of PNAISM allowed menopausal women to have access to health care and was an incentive to the family health care strategy teams to act at the level of public policy, orienting the promotion, prevention, and health actions in all Brazilian territories [12, 25]. It was also a stimulus for family health care strategy policies aimed at controlling chronic noncommunicable diseases. Both may have contributed to the reduction in mortality from circulatory system diseases [20–25].

In the course covered by the 20 years of this study (1996–2016), the mortality rates due to neoplasms remained constant compared to the other major causes of death. Furthermore, mortality from neoplasms did not increase among menopausal women in Brazil, according to our results. This was a scenario that maintained its stability independent of and subsequent to the implementation of PNAISM (2005–2016).

Implementation of public policies aimed at an early diagnosis and treatment of cancer—such as the National Cancer Care Policy (Política Nacional de Atenção Oncológica) in 2005, along with the Greater Health Program (Programa Mais Saúde) in 2009, and the National Plan for a Stronger Network for Cancer Prevention, Diagnosis, and Treatment (Plano Nacional de Fortalecimento da Rede de Prevenção, Diagnóstico e Tratamento do Câncer) in 2011— diminished the cancer load in Brazil [29–32].

The stability of the mortality rates for neoplasms in the post-PNAISM period detected in the present study may be regarded as an expected result, given the cancer progression time from early detection to the time of death with respect to breast [29] and cervical [30] cancers as well as an improvement in the filling out of forms and in the quality of information of the information systems concerning the causes of death [29–31]. Still, the stability of the cancer mortality rates may be viewed as a warning for the type of unorganized screening performed in Brazil, with breast and cervical cancers at the forefront in women’s health [32].

There was no reduction in deaths from respiratory diseases, a finding also shown in the results of Schmitt et al. [8] The implementation success of other public policies, such as that of smoking [33], could have changed the outcome. However, it is worth highlighting that, despite the studies [34, 35], which demonstrate the harmful effects of environmental pollution, the behavioral changes in women who have been smoking less in the recent decades, and the promotion of smoking cessation campaigns, mortality from respiratory diseases has not decreased.

The reduction in mortality from endocrine, nutritional, and metabolic diseases following PNAISM implementation, as shown in this study as well as in mortality from circulatory system diseases, may be associated with a reduction in shared risk factors, such as diabetes mellitus and dyslipidemia [36], and with the health care provided to (Brazilian) diabetic patients, by making available pharmacological treatments (oral hypoglycemic drugs and insulin) through the SUS [36].

The mortality analysis of neoplasms as a whole and not as separate entities—some of which are the top priority list of women’s health care in PNAISM (particularly cervical and breast cancers)—was a limitation of the present study. However, PNAISM should reduce morbidity and mortality due to neoplasms in general. In addition, the implementation of National Health Policies does not clearly place health indicators or targets related to health parameters. The analysis of its results was limited by: a) the lack of ascertainment of menopause status, which was poor and inconsistent in the DATASUS database; b) the ecologic design which only correlates the mortality rates to time periods before and after the PNAISM so that other factors unrelated to implementation of the PNAISM may have changed during these time periods which could account for any changes so that any attribution of causality was inappropriate; c) the current analyses did not include adjustment for any potential confounding factors such as smoking, body mass index, family history or exposure to air pollution, which may have also changed over the time period studied but they could not be extracted in the vast period analysed in the study.. In relation to this point, it should be considered that the age of women in our analyses was between 40 and 64 years old, which was less than the usual age of mortality among women in our country.

What is novel about this study is that it acknowledges PNAISM as a public health milestone and as a wellspring of thought more than two decades after its implementation. This national policy is one of the pillars in SUS [37, 38] of the maintenance and assurance of actions for prevention and promotion that impact women’s mortality, specifically during the climacteric period. Thus, analyses of women’s health indicators, such as mortality rates and health diagnoses, are necessary and should make it possible to monitor the benefits of PNAISM, taking into account health care levels and the female life cycle [39].

## Conclusions

The primary causes of death among climacteric women were: 1- circulatory system diseases; 2- neoplasms; 3- respiratory system diseases; 4- endocrine, nutritional, and metabolic diseases; and 5- digestive system diseases. After implementation of the PNAISM, we observed a downward trend in rates of mortality from diseases of the circulatory and digestive systems and from endocrine, nutritional, and metabolic diseases but stability in the rates of death from neoplasm and respiratory system diseases. The analysis of women’s health indicators, such as mortality rates, is fundamental to enable the monitoring of benefits and results related to PNAISM as well as directing the design and implementation of other new health policies to be developed for women.

## Data Availability

The participant or patient data was de-identified. The datasets used and/or analysed during the current study are publically available in the DATASUS repository: https://datasus.saude.gov.br/mortalidade-desde-1996-pela-cid-10.

## Abbreviations

PAISM: Política de Atenção Integral à Saúde da Mulher
PNAISM: Política Nacional de Atenção Integral à Saúde da Mulher
UHS: Unified Health System
SUS: Sistema Único de Saúde
DATASUS: IT Department of SUS
IBGE: Instituto Brasileiro de Geografia e Estatística
SIM: Sistemas de Informação sobre Mortalidade
ICD-10: International Classification of Diseases-10
*R*^2^: Coefficient of determination
CI: Confidence interval
